# Rehabilitation of Older Asian Traumatic Brain Injury Inpatients: A Retrospective Study Comparing Functional Independence between Age Groups

**DOI:** 10.3390/life13102047

**Published:** 2023-10-13

**Authors:** Rathi Ratha Krishnan, Samuel Wen Xuan Ting, Wee Shen Teo, Chien Joo Lim, Karen Sui Geok Chua

**Affiliations:** 1 Department of Rehabilitation Medicine, Tan Tock Seng Hospital Rehabilitation Centre, Singapore 307382, Singapore; 2 Lee Kong Chian School of Medicine, Nanyang Technological University, Singapore 308232, Singapore; 3 Yong Loo Lin School of Medicine, National University of Singapore, Singapore 117597, Singapore; 4 Institute of Rehabilitation Excellence, Tan Tock Seng Hospital Rehabilitation Centre, Singapore 307382, Singapore; 5 Department of Orthopaedic Surgery, Woodlands Health, Singapore 737628, Singapore

**Keywords:** traumatic brain injury, rehabilitation, age, geriatric, head injury

## Abstract

Across traumatic brain injury (TBI) severities, a geriatric TBI tsunami has emerged. Mixed outcomes are reported for elderly TBI with positive functional improvements with acute inpatient rehabilitation. We studied the effect of age at TBI on discharge functional outcomes, levels of independence and length of stay. A retrospective analysis of Asian TBI patients during inpatient rehabilitation over a 4-year period was conducted. Independent variables included admission GCS, post-traumatic amnesia (PTA) duration and injury subtypes. Primary outcomes were discharge Functional Independence Measure (Td-FIM) and FIM gain. In total, 203 datasets were analysed; 60.1% (122) were aged ≥65 years (older), while 39.9% (81) were <65 years (younger). At discharge, older TBI had a significantly lower Td-FIM by 15 points compared to younger (older 90/126 vs. younger 105/126, *p* < 0.001). Median FIM gains (younger 27 vs. older 23, *p* = 0.83) and rehabilitation LOS (older 29.5 days vs. younger 27.5 days, *p* = 0.79) were similar for both age groups. Older TBIs had significantly lower independence (Td-FIM category ≥ 91) levels (49.4% older vs. 63.9% younger, *p* = 0.04), higher institutionalisation rates (23.5% older vs. 10.7% younger, *p* = 0.014) and need for carers (81.5% older vs. 66.4% younger, *p* = 0.019) on discharge. Although 77% of older TBI patients returned home, a significantly higher proportion needed care. This study supports the functional benefits of TBI rehabilitation in increasing independence regardless of age without incurring longer inpatient rehabilitation days.

## 1. Introduction

Traumatic brain injury (TBI) is a significant source of morbidity and mortality worldwide [1,2,3], with the incidence of TBI estimated at 69 million [4] per year. In Singapore, trauma constitutes the fifth principal cause of death [5]. Falls and road traffic accidents (RTAs) make up the two predominant causes of TBI-related hospitalisation [6].

There is an increasing global incidence of TBI starting at about 70 years, with falls overtaking RTAs as the main cause [7]. Compared to a decade ago, the demographics of TBI patients have shifted rapidly towards an increasingly older population due to world population ageing and longer life expectancies [8]. Concomitant antiplatelet and anticoagulation medication use among the elderly can further worsen TBI severity with relatively mild trauma. Preventative measures, such as seat belt laws, speed controls and vehicle design, have also reduced the incidence of severe TBIs related to RTAs, which are more common in younger ages, while falls have overtaken RTAs as the main cause of TBIs [9].

Older age has often been identified as a risk factor for poorer outcomes in TBI [10,11,12,13,14], regardless of injury severity or mechanism [15,16,17]. Furthermore, elderly patients generally require longer hospital stays [18,19] and are more likely to experience late neurologic decline [20,21]. Unfortunately, this has also created a bias towards the differential treatment of older TBI compared with younger TBI, favouring a more conservative approach due to higher co-morbidities and frailties. In a multi-centre prospective study by Munro et al. in Scotland, it was noted that older patients with closed head injuries were less likely to be referred for specialist neurosurgical interventions compared to their younger counterparts. There was also a clear difference noted in the process of care between the older and younger patients. The authors concluded that this might be due to doctors’ concerns over poorer functional outcomes and survival rates in older patients based on previous studies, resulting in scepticism over the worth of treating older patients with TBI [22]. 

However, there has been a shift in the perspective amongst clinicians that functional and cognitive outcomes in elderly cases of TBI may be as good as TBIs in younger people if sufficient resources, timely interventions and aggressive rehabilitation are provided. Mak et al. concluded that though the elderly fared worse after a TBI, those aged 65–75 years could have a comparable outcome to younger adults [23].

Countries in Asia are ageing more rapidly compared to Europe and North America [24]. In an epidemiological study by Liew et al., a cohort of local TBI patients admitted to a single tertiary medical institution were retrospectively analysed. Their study concluded that the demographics of TBI patients have moved to an older population, with an increased incidence of falls [8]. Despite the emergence of a geriatric TBI tsunami in Asia, there have been few Asian studies describing their rehabilitation outcomes. Liew’s paper stated that TBIs amongst the elderly, compared to younger people, had poorer outcomes and longer hospitalisation stays. The elderly also had lower rates of functional improvement on discharge, as measured on the Glasgow Outcome Scale (GOS). In contrast, Yap et al. [25], in 2008, found that age was not a predictor of outcome in TBI patients, with the elderly experiencing significant functional gains as measured on the Modified Barthel Index (MBI) and high rates of community discharge.

We conducted a retrospective medical records review of inpatient TBIs to primarily compare functional outcomes between TBIs in younger and older patients and, secondarily, to study the impact of demographic, clinical TBI characteristics on discharge function using the Functional Independence Measure (FIM)^®^ For this study, we used the age cut-off of 65 years to define the older patients based on WHO definitions [26]. Our study has potential implications for the healthcare resources for the rehabilitation of older TBI survivors in view of the ageing of the world’s population [27].

## 2. Materials and Methods

### 2.1. Study Design

A retrospective analysis of discharged electronic medical records (EMRs) of patients from 1 January 2016 to 31 December 2019 who underwent and completed inpatient TBI rehabilitation at Tan Tock Seng Hospital (TTSH) Rehabilitation Centre, Singapore, was conducted. Institutional ethics approval was obtained prior to data collection (NHG-DSRB 2020/00298). Consent was waived as no humans were directly involved, and no patient identifiers were collected. 

### 2.2. Study Setting

The study was conducted in a single tertiary rehabilitation unit of a level II trauma centre with direct links to an acute neurosurgical unit. Patients are transferred to rehabilitation after screening by physiatrists. The consultant-led inpatient TBI rehabilitation program consists of rehabilitation therapies delivered for 3 h daily over 5.5 days a week by a multidisciplinary team of physiotherapists, occupational therapists, speech pathologists, nurses, social workers and psychologists. FIM was recorded within 72 h of admission and discharge of rehabilitation by trained rehabilitation therapists.

### 2.3. Study Population

Study inclusion criteria were: (i) first-time TBI patients diagnosed by neurosurgeons and confirmed on CT or MRI brain imaging, (ii) aged 21–85 years and of Asian ethnicity, (iii) admitted to rehabilitation within 6 months of TBI, and (iv) completed inpatient rehabilitation. 

Study exclusion criteria were: (i) non-TBI diagnosis (e.g., stroke, tumour), (ii) previous TBI, (iii) non-Asian ethnicity and (iv) incomplete FIM scores.

### 2.4. Data Collection Procedure, Variables and Outcome Measures

A case record form was constructed based on pseudonymised data extracted from NHG-CDOC records to a hard copy data collection form, which was then entered into the NHG REDCap platform and exported to MS Excel (Version 2309).

The following independent acute variables included: (i) demographic characteristics including age, gender, race, employment status and co-morbidities; (ii) TBI injury mechanisms: road traffic accident, fall, assault, sport, struck by object, occupational, others; (iii) acute TBI characteristics: admission Glasgow Coma Scale (GCS) [28,29], acute length of stay (LOS), coma duration (days), ICU stay, neurosurgical interventions, such as craniotomy, intracranial pressure monitors, ventriculoperitoneal shunts, decompressive craniectomy and the presence of seizures.

The following independent rehabilitation variables included: length of stay in rehabilitation-RLOS (days), (ii) motor impairment, (iii) post-traumatic amnesia (PTA), (iv) complications during rehabilitation defined as those requiring treatment or disrupting rehabilitation (e.g., urinary tract infections, decubitus ulcers) and (v) numbers and types of transfers back into acute care due to medical or surgical complications. PTA refers to a state of confusion and disorientation following TBI and the duration of PTA was measured using the Westmead Post-traumatic Amnesia Scale (WMPTAS) [30]. PTA testing is performed by the occupational therapist in the TTSH Rehabilitation Centre. The patient must have regained consciousness and be able to communicate via speech, writing, pointing to answers or indicating yes or no. PTA test is conducted in a quiet environment with no distractions. PTA testing is stopped when a patient has achieved a full score of 12 on the WMPTAS for three consecutive days. 

Dependent rehabilitation variables included (i) admission and discharge functional using the FIM^®^ [31,32] with a score ranging from 18–126; (ii) discharge disposition classified as home, community hospital or nursing home; (iii) whether a caregiver was required.

FIM is an ordinal scale used to determine the degree of disability and the progress that patients make through medical rehabilitation. It has two components: motor FIM with a sub-score of 91 and cognitive FIM with a sub-score of 35.

The primary outcome measure for this study was total discharge FIM (Td-FIM). Secondary outcome measures included FIM gain (FIM-G) calculated as total admission FIM (Ta-FIM)—total discharge FIM (Td-FIM). FIM efficiency refers to FIM-G/LOS in rehabilitation. Both these quantify, respectively, the absolute gain and aggregated rate of progress in rehabilitation.

### 2.5. Statistical Analysis

Statistical analysis was carried out with IBM Statistical Package for the Social Sciences (SPSS version 27). Descriptive statistics were used to present the characteristics of the patients. Data were assessed for normality with the Shapiro–Wilk test. Data were presented as mean and standard deviation (SD) if the data were normally distributed, the median and interquartile range (IQR). Categorical variables were presented as frequency and percentage.

The two-tailed independent sample T-test, Mann–Whitney U test, Pearson chi-squared test and the Fisher Exact test were used to compare the characteristics between age groups. At the same time, the Wilcoxon signed-rank test was used to explore the change in FIM scores during rehabilitation. A correlation matrix and multiple linear regression analyses were performed to determine factors predictive of Td-FIM. The level of significance was set at *p* < 0.05.

## 3. Results

### 3.1. Baseline Data

In all, 598 datasets were screened from the brain injury database, and 203 datasets were available for analysis. A total of 395 patients were excluded for the following reasons: non-TBI (293) had a non-TBI, previous TBI (58), incomplete FIM data (25), admitted >6 months post TBI (15), did not complete inpatient rehabilitation (4). 

### 3.2. Comparison of Acute and Rehabilitation Characteristics between Groups

Table 1 shows the comparison of acute demographic and injury characteristics by age group. 

Younger patients < 65 years comprised 60.1% (122) of the population, while elderly patients ≥65 years comprised 39.9% (81) of the population. The majority of the elderly were unemployed/retired (7.4% younger vs. 77.8% elderly, *p* < 0.001).

The elderly had significantly higher rates of co-morbidities (67.2% younger vs. 90.1% elderly, *p* < 0.001). Falls were the predominant cause of TBI (50.7%), with a significantly higher proportion occurring in the elderly (29.5% younger vs. 82.7% elderly, *p* < 0.001). A large proportion of the elderly had a mild TBI on admission, as indicated by a GCS of 13–15 (26.2% younger vs. 71.6% elderly, *p* < 0.001).

Table 2 shows rehabilitation characteristics by age group. PTA data were available for 77.8% of the population. Reasons for absent PTA scores included inattention, agitation, aphasia or ineligibility to use the Westmead PTA scale (e.g., patients with a premorbid diagnosis of dementia).

Duration of PTA for older population was significantly longer by 3 days compared to younger (34 days younger vs. 37 days elderly, *p* < 0.001). Both age groups were comparable in their ability to emerge from PTA (74.2% younger vs. 63.2% elderly, *p* = 0.189).

During rehabilitation, both groups had similar rates of complications (55.7% younger vs. 58.0% elderly, *p* = 0.747).

Both age groups had comparable LOS in rehabilitation (27.5 days in younger vs. 29.5 days in elderly, *p* = 0.786). However, the elderly were significantly more likely to be institutionalised at discharge from rehabilitation (10.7% younger vs. 23.5% elderly, *p* = 0.014), with more requiring carers (66.4% younger vs. 81.5% elderly, *p* = 0.019).

### 3.3. FIM Comparisons

Table 3 shows the total progress of FIM scores over time. This was significant across all domains of Total, Motor and Cognitive FIM from admission to discharge (*p* < 0.001).

Table 4 shows the comparison of FIM scores by age group. 

On admission, there were no significant differences for Ta-FIM, motor (M-FIM) and cognitive (C-FIM) between both age groups. 

At discharge, there was a 15-point FIM difference between older (younger, 105 vs. older, 90, *p* < 0.004); the majority of this difference was contributed by gains in motor-FIM (younger M-FIM 78 vs. elderly M-FIM 67, *p* < 0.001), while cognitive-FIM was similar between age groups (younger C-FIM 27 vs. elderly C-FIM 26, *p* = 0.106). FIM gain and efficiency were not significantly different. Furthermore, significantly fewer elderly (49.4% elderly vs. 63.9% younger, *p* = 0.04) needed no career on discharge (FIM ≥ 91 indicating modified independence on all components of FIM).

### 3.4. Variables Impacting Td-FIM

Table 5 shows a multiple regression model for variables impacting Td-FIM. Variables were selected based on their significance in other literature [8,10,33] or through statistically significant variables in a bivariate correlation model. R2 was 0.71, indicating a strong effect size.

Positive relationships were noted between Td-FIM with pre-injury employment, rehabilitation LOS, admission M-FIM and PTA emergence. Those who had retired or were unemployed were observed to have 11.55 points lower Td-FIM than those who were studying or working (*p* = 0.003), while emergence from PTA correlated with a gain of 15.7 points (*p* < 0.001) in Td-FIM, but not the duration or severity of PTA. Negative relationships were noted between Td-FIM with longer RLOS (−0.18, *p* < 0.001) and craniectomy (−7.7 points, *p* = 0.008). 

Notably, older age (*p* = 0.732), GCS severity (mild *p* = 0.802; severe *p* = 0.857) and admission C-FIM (*p* = 0.983) did not affect Td-FIM.

## 4. Discussion

### 4.1. TBI Epidemiology

The epidemiology of TBI patients has shifted rapidly over the last decade. A local TBI cohort in 2006 [6] reported a mean age of 44.6 years in comparison to our sample’s mean of 58.5 years. This increase in the mean age of TBI patients in the rehabilitation centre may be attributed to a few factors. One, the ageing population in Singapore, coupled with improved healthcare, leads to higher life expectancy [34]. Another factor may be reduced mortality amongst elderly TBI patients due to improved acute TBI management. The trend of increasing incidence of TBI amongst the elderly has been noticed in the United States as well as other high-income countries across the globe [35]. We do not have a TBI registry in Singapore, only a National Trauma Registry. According to the TBI Report by the Centers for Disease Control and Prevention (CDC) in the United States of 2002–2006, large increases were noted in emergency department visits by the elderly aged 65 years and above. It was postulated that the increased visits could be attributed to an increased public awareness of TBI. In this study, falls were noted to be the major cause of TBI amongst the elderly. This underlies the need to seriously look into fall prevention among the elderly. The current emphasis of fall prevention programmes in Singapore is on the prevention of fragility fractures. Perhaps attention could also be given to the prevention of head injuries. 

### 4.2. Comparison of Functional Outcomes by Age Group

This study demonstrated significant functional benefits after inpatient specialised TBI rehabilitation for both young and elderly populations, with significant gains within groups in Td-FIM. However, it was noted that younger TBI individuals tended to attain minimal clinically important difference (MCID) thresholds for FIM compared to the older TBI, i.e., FIM-G 27 for TBI [36,37] (median FIM-G: 27 young vs. 23 elderly, *p* = 0.083) without significantly different RLOS by age. 

Despite the majority of the elderly having mild injuries (GCS 13–15, older 71.6% vs. younger 26.2%), shorter acute stays by one week and lower craniotomy rates, 73.7% had >28 days of PTA consistent with very severe TBI [38]. It should also be noted that though GCS is widely used to assign the severity of TBI, it is poorly predictive of morbidity and mortality in older adults, who tend to present with better initial GCS scores than younger people with the same injury severity [35].

Furthermore, TBI in the elderly resulted in worse outcomes, with a 15-point lower Td-FIM compared to younger patients and more needed carers on discharge due to lower independence rates (FIM < 91). Motor, rather than cognitive, FIM gains were significantly lower by 11 FIM points in older vs. younger TBI, despite worse TBI in the young as evidenced by higher proportions with lower admission GCS, surgeries and longer acute stays. This could be explained by differential injury mechanisms, with a predominance of RTAs in younger patients (57.4% young vs. 11.1% elderly) and low-velocity falls [39] in the elderly (82.7% elderly vs. 29.5% young). Other reasons include neuromotor and functional emphases during inpatient rehabilitation compared to cognitive rehabilitation, usually deferred to PTA emergence post-discharge [33]. 

On discharge, older TBI patients achieved lower Td-FIM, needed care and had higher institutionalisation rates by 2.3× compared to their younger peers. Previous studies have documented older age, living alone before TBI and lower levels of function at rehabilitation discharge as predictors for institutionalisation [40]. Other studies have also found similar trends in elderly functional outcomes post-rehabilitation [41].

That said, the elderly population demonstrated efficiency during rehabilitation with comparable rehabilitation LOS, similar FIM gains and efficiency. This is in contrast to earlier studies, which suggested that elderly patients required 3× longer in rehabilitation to make similar gains than younger TBI patients [18,42,43]. This is significant as a local study found that rehabilitation LOS is a main contributor to the direct inpatient hospital cost and could suggest that inpatient rehabilitation of the elderly may have similar direct costs as younger patients [44]. 

There were high rates of home discharge (76.5%) for the elderly, comparable to that seen in Frankel and Cifu’s studies (80% and 82% discharged home, respectively) [42,43]. Of note, these two authors defined older TBI as >55 years of age. Other studies have also advocated for the aggressive acute treatment and rehabilitation of elderly TBI patients [23,45,46,47].

### 4.3. Factors Affecting Td-FIM

Our regression model found impactful premorbid, acute injury and rehabilitation variables. The age group was not significant. Premorbid employment was a significant variable, with employed individuals having +11.6 points higher Td-FIM compared to unemployed individuals, suggesting that higher cognitive or functional reserves offered by meaningful work were important in rehabilitation outcomes.

Patients undergoing decompressive craniectomy (DC) had −7.7 points on Td-FIM. This was not surprising as DC is a procedure reserved as a last-resort therapy for patients with refractory raised ICP [48]. These patients had severe injuries and, thus, worse outcomes.

Of importance was the ability to emerge from PTA. Patients who emerged from PTA gained +16.5 in FIM score compared to those who did not. In contrast, PTA duration was not a significant predictor of Td-FIM. This is a notable finding, as current literature has not linked PTA emergence to rehabilitation discharge outcomes. This suggests further potential for research into specific strategies to accelerate PTA emergence.

Patients aged between 75 to 84 years were also noted to have significantly lower rates of outpatient injury-related clinic visits and significantly higher rates of rehospitalisation, home health care visits and weekly hours of unpaid care from friends and family compared with patients aged 55 to 74 years, suggesting possible age-related disparities in coordinated care after hospital discharge [49]. Hence, there is an inevitable need to develop clear, evidence-based guidelines for managing the growing population of elderly TBI patients to improve their overall outcomes. Having neurorehabilitation in the trauma centre early, followed by more intensive neurorehabilitation in the rehabilitation facility, has been shown to improve the functional recovery of patients with moderate to severe TBI compared with usual care [50].

We acknowledge the limitations of our study. Firstly, it was a retrospective, single-centre study with a relatively small sample size; thus, our findings may have limited generalisability to the general TBI population or TBI aged >80 years, as none of our patients were octogenarians. Our study may also contain a selection bias due to the assessing physicians prior to entry to inpatient rehabilitation.

## 5. Conclusions

This study supports the functional benefits of acute TBI rehabilitation in increasing independence in the older TBI regardless of age at presentation. The elderly were able to achieve significant functional gains and independence levels comparable to their younger counterparts without incurring longer inpatient rehabilitation days. The majority were able to be discharged back into the community, albeit with more older TBI patients needing care.

Given that we are in the midst of a global geriatric TBI tsunami, population health measures are needed to drive TBI prevention aggressively for the elderly in relation to fall prevention, reducing sarcopenia frailty and making homes and outdoor environments safer. Adherence to elderly acute TBI protocols and early intensive rehabilitation is evidence-based management.

## Figures and Tables

**Table 1:** Comparison of demographic, injury and acute TBI characteristics by age group (n = 203).

Variable	Total (n = 203)	Age < 65 y (n = 122)	Age ≥ 65 y (n = 81)	*p*-Value
Age				
Age in years, mean (SD)	58.5 (19.8)	41.42 (14.99)	73 (5.7)	<0.001 ^a^
Gender, n (%)				
Male	160 (78.8)	98 (80.3)	62 (76.5)	0.518 ^c^
Female	43 (21.2)	24 (19.7)	19 (23.5)	
Race, n (%)				
Chinese	152 (74.9)	79 (64.8)	73 (90.1)	<0.001 ^c^
Non-Chinese	51 (25.2)	43 (35.2)	8 (9.9)	
Malay	27 (13.3)	24 (19.7)	3 (3.7)	
Indian	19 (9.4)	14 (11.5)	5 (6.2)	
Others	5 (2.5)	5 (4.1)	0 (0.0)	
Employed at time of TBI, n (%)				
No	72 (35.5)	9 (7.4)	63 (77.8)	<0.001 ^b^
Yes	131 (64.5)	113 (92.6)	18 (22.2)	
Pre-Injury Comorbidities, n (%)				
Absent	48 (23.6)	40 (32.8)	8 (9.9)	<0.001 ^c^
Present	155 (76.4)	82 (67.2)	73 (90.1)	
Injury mechanism, n (%)				
Road traffic accident	79 (38.9)	70 (57.4)	9 (11.1)	<0.001 ^c^
Fall	103 (50.7)	36 (29.5)	67 (82.7)	<0.001 ^c^
Others	21 (10.3)	16 (13.1)	5 (6.2)	<0.001 ^d^
GCS on acute admission, n (%)				
Mild (13–15)	90 (44.3)	32 (26.2)	58 (71.6)	<0.001 ^c^
Moderate (9–12)	52 (25.6)	39 (32.0)	13 (16.0)	0.011 ^c^
Severe (3–8)	61 (30.0)	51 (41.8)	10 (12.3)	<0.001 ^c^
ICU admission, n (%)				
Absent	63 (31.0)	32 (26.2)	31 (38.3)	
Present	140 (69.0)	90 (73.2)	50 (61.7)	0.069 ^c^
Neurosurgery, n (%)				
Not required	75 (36.9)	43 (35.2)	32 (39.5)	
Required	128 (63.1)	79 (64.8)	49 (60.5)	0.513 ^c^
Types of procedures				
ICP monitor	61 (30.0)	47 (38.5)	14 (17.3)	0.001 ^c^
Decompressive craniectomy	39 (19.2)	30 (24.6)	9 (11.1)	0.017 ^c^
EVD	27 (13.3)	18 (14.8)	9 (11.1)	0.454 ^c^
Evacuation of clot	39 (19.2)	20 (16.4)	19 (23.5)	0.211 ^c^
Craniotomy	30 (14.8)	13 (10.7)	17 (21.0)	0.42 ^c^
Tracheostomy	17 (8.4)	10 (8.2)	7 (8.6)	0.911 ^c^
VP shunt	8 (3.9)	6 (4.9)	2 (2.5)	0.481 ^d^
Post TBI seizure, n (%)				
Absent	182 (89.7)	104 (85.2)	78 (96.3)	0.011 ^c^
Present	21 (10.3)	18 (14.8)	3 (3.7)	
Acute LOS (days), median (IQR)	23 (23)	25 (25)	18 (18)	<0.001 ^b^

^a^ Independent Samples *t* test; ^b^ Mann–Whitney U test; ^c^ Pearson Chi Square test; ^d^ Fisher Exact test (Legend: TBI: Traumatic Brain Injury, GCS: Glasgow Coma Scale, ICU: Intensive Care Unit, ICP: Intracranial Pressure Monitor, VP: Ventricular Peritoneal, LOS: Length of stay, IQR: interquartile range).

**Table 2:** Comparison of rehabilitation characteristics by age group (n = 203).

Variable	Total (n = 203)	Age < 65 y (n = 122)	Age ≥ 65 y (n = 81)	*p*-Value
PTA Duration in days, median (IQR)	35 (33)	34 (35)	37 (30)	<0.001 ^b^
PTA ≥ 28 days, n (%)	107 (67.7)	65 (64.4)	42 (73.7)	0.228 ^a^
PTA emergence by discharge, n (%)	111 (70.2)	75 (74.2)	36 (63.2)	0.189 ^a^
Motor impairment, n (%)				
Absent	147 (72.4)	87 (71.3)	60 (74.1)	0.672 ^a^
Present	48 (23.6)	31 (25.4)	17 (21.0)	
Unable to assess (n = 195)	8 (3.9)	4 (3.3)	4 (4.9)	
LOS rehabilitation (days), median (IQR)	28.5(28)	27.5 (32)	29.5 (26)	0.786 ^b^
Medical complications, n (%)				
Present	115 (56.7)	68 (55.7)	47 (58.0)	0.747 ^a^
Absent	87 (42.9)	53 (43.4)	34 (42.0)	
Transfer out, n (%)				
Present	30 (14.8)	16 (13.1)	14 (17.3)	0.412 ^a^
Absent	173 (85.2)	106 (86.9)	67 (82.7)	
Discharge destination, n (%)				
Home	171 (84.2)	109 (89.3)	62 (76.5)	0.014 ^a^
Institution/Others	32 (15.8)	13 (10.7)	19 (23.5)	
Carer needed, n (%)				
Yes	146 (71.9)	81 (66.4)	66 (81.5)	0.019 ^a^
No	56 (27.6)	41 (33.6)	15 (18.5)	

^a^ Pearson Chi Square test; ^b^ Mann–Whitney U test (Legend: PTA; Post traumatic amnesia, LOS: Length of stay, IQR: interquartile range).

**Table 3:** Group summary table on changes in FIM during rehabilitation (n = 203).

Variables	Admission	Discharge	*p*-Value
Total FIM, median (IQR)	62.0 (49.0)	96.5 (38.0)	<0.001
Motor FIM, median (IQR)	42.0 (36.0)	73.0 (28.0)	<0.001
Cognitive FIM, median (IQR)	18.0 (17.0)	26.0 (12.0)	<0.001

Wilcoxon Signed Rank test (Legend: FIM: Functional Independence Measure, IQR: interquartile range).

**Table 4:** Comparison of FIM scores by age category by time point (n = 203).

Variable	Total (n = 203)	Age < 65 y (n = 122)	Age ≥ 65 y (n = 81)	*p*-Value
FIM (admission)				
Total FIM, median (IQR)	62 (49)	63 (52)	62 (47)	0.570 ^a^
Motor FIM, median (IQR)	42 (36)	46 (38)	40 (31)	0.234 ^a^
Cognitive FIM, median (IQR)	18 (17)	17 (17)	19 (14)	0.302 ^a^
FIM (discharge)				
Total FIM, median (IQR)	96.5 (38)	105 (37)	90 (41)	0.004 ^a^
Motor FIM, median (IQR)	73 (28)	78 (25)	67 (32)	<0.001 ^a^
Cognitive FIM, median (IQR)	26 (12)	27 (12)	26 (13)	0.106 ^a^
Calculated scores				
FIM gain, median (IQR)	26 (25.5)	27 (32)	23 (26.5)	0.083 ^a^
FIM efficiency, median (IQR)	0.886 (1.13)	1.07 (1.29)	0.8 (1.03)	0.131 ^a^
FIM ≥ 91, number (%)	118 (58.1)	78 (63.9)	40 (49.4)	0.040 ^b^
FIM < 91, number (%)	85 (41.9)	44 (36.1)	41 (50.6)	

^a^ Mann Whitney U test, ^b^ Pearson Chi Square test (Legend: FIM: Functional Independence Measure, IQR: interquartile range).

**Table 5:** Multiple linear regression analysis on factors impacting total discharge FIM (n = 203).

Variables	Multiple Linear Regression		
	Adj. Coeff	95% CI	*p*-Value
Age			
<65 y	Ref		
≥65 y	−1.20	−8.10, 5.70	0.732
Employment			
Studying or working	Ref		
Retired or unemployed	−11.55	−18.84, −4.26	0.002
GCS severity			
Mild	Ref		
Moderate	−0.75	−6.66, 5.15	0.802
Severe	−0.58	−6.89, 5.73	0.857
Decompressive craniectomy	−7.69	−13.38, −2.01	0.008
Craniotomy	−5.27	−12.17, 1.64	0.134
Length of stay at acute hospital	−0.04	−0.20, 0.11	0.588
Length of stay at rehab	−0.18	−0.27, −0.10	<0.001
Motor FIM on admission	0.50	0.32, 0.69	<0.001
Cognitive FIM on admission	−0.00	−0.32, 0.31	0.983
PTA emergence on discharge	16.51	10.99, 22.04	<0.001
PTA > 28 days	−4.26	−10.00, 1.47	0.144

Variable selection enter method was used; R^2^ = 0.709. R^2^ is the percentage of total variance explained by the model (Legend: GCS: Glasgow Coma Scale, FIM: Functional Independence Measure, PTA: Post-Traumatic Amnesia).

## Data Availability

The data presented in this study are not publicly available due to privacy reasons.
